# RPN2 promotes colorectal cancer cell proliferation through modulating the glycosylation status of EGFR

**DOI:** 10.18632/oncotarget.20005

**Published:** 2017-08-07

**Authors:** Haiping Li, K Al-Japairai, Yong Tao, Zheng Xiang

**Affiliations:** ^1^ Department of Gastrointestinal Surgery, The First Affiliated Hospital of Chongqing Medical University, Chongqing, China; ^2^ Chongqing Key Laboratory of Department of General Surgery, The First Affiliated Hospital of Chongqing Medical University, Chongqing, China

**Keywords:** colorectal cancer, RPN2, EGFR, proliferation, glycosylation

## Abstract

Various studies have found that silencing ribophorin II (RPN2) inhibits cell growth in several cancers. However, the underlying mechanism by which RPN2 regulates cancer cell proliferation remains unclear. Herein, we reveal that downregulation of RPN2, which may be a crucial regulator of N-linked glycosylation in cancer cells and drug-resistant cancer cells, promoted the progression of colorectal cancer (CRC) cell cycle and proliferation *in vitro* and *in vivo*. We found that RPN2 silencing reduced glycosylation of EGFR, a highly N-link glycosylated cell surface glycoprotein that plays a critical role in majority of human cancers correlating with increased cell growth, proliferation, and differentiation. In addition, RPN2 knockdown decreased EGFR expression and cell surface transport by EGFR deglycosylation. In summary, our findings suggest that RPN2 regulates CRC cell proliferation through mediating the glycosylation of EGFR which affecting the EGFR/ERK signaling pathways. Clinicopathological analysis showed that the overexpression of RPN2 and EGFR was positively correlated with colorectal tumor size. Therefore, RPN2 may be a new therapeutic target and prognostic biomarker for CRC.

## INTRODUCTION

CRC is one of the most common malignant tumors, with more than 1.2 million patients diagnosed each year, and more than 600,000 annual deaths [1]). Although much effort has focused on probing the pathogenesis of the disease, the molecular mechanisms underlying the process are still unclear [2].

RPN2 is a highly conserved glycoprotein located exclusively in the membranes of the rough endoplasmic reticulum (ER) and involved in the translocation and maintenance of the structural uniqueness of the rough ER [3, 4]. Previous research has demonstrated that the RPN2 protein is a part of an oligosaccharyltransferase (OST) complex that conjugates high mannose oligosaccharides to asparagine residues in the N-X-S/T consensus motif of nascent polypeptide chains [5, 6]. Furthermore, studies have shown that RPN2 knockdown can inhibit cancer cell proliferation in osteosarcoma [7], non-small cell lung cancer [8, 9], and breast cancer [10]. Inhibition of RPN2 expression was found to reduce breast cancer malignancy by reducing CD63 glycosylation and modulating translocation of CD63 [10], which is a cell surface glycoprotein with N-linked glycosylation that regulates cell motility, invasion, and cell signaling of tumors [11]. Analogously, RPN2 knockdown induced docetaxel-dependent apoptosis and cell growth inhibition in human breast cancer cells by reducing glycosylation of the P-glycoprotein with N-linked glycosylation, as well as decreasing membrane localization [12]. These studies revealed that RPN2 is a crucial regulator of N-linked glycosylation in cancer cells and drug-resistant cancer cells. Integrated transcriptional profiling and genomic analyses have revealed that RPN2 is a promising biomarker in CRC [13]. However, the correlation of RPN2 and CRC malignancy, as well as the specific mechanisms, is still poorly understood.

EGFR is a highly N-linked glycosylated cell surface glycoprotein [14] that plays a critical role in the majority of human cancers correlating with increased cell growth, proliferation, and differentiation [15, 16]. An increasing number of studies have indicated the importance of N-glycosylation on the functional properties of EGFR, including cell surface expression [17, 18], ligand binding [19], conformational stability [20], dimerization [21], interaction with membranes [22], and endocytosis [23]. Early studies showed that N-glycosylation of the ectodomain of EGFR contributes about 40 kDa of the mass to the 175 kDa mature protein [24, 25]. Meanwhile, sequence analysis showed that there are 11 potential N-glycosylation sites in the extracellular domain of the EGFR [26]. In addition, in the presence of tunicamycin, an inhibitor of N-linked glycosylation, a 130-135 kDa immature EGFR protein is synthesized, which apparently does not reach the cell surface [24] and does not acquire the capacity to bind EGF as measured by binding to an EGF affinity matrix or by a soluble binding assay [27]. However, little attention has been focused on the contribution of N-glycosylation of EGFR to CRC malignancy.

To the best of our knowledge, no studies have examined the correlation between RPN2 and specific N-linked glycoproteins that are correlated with CRC malignancy. Herein, we found that CRC cell cycle progression was blocked in the G1-S phase and proliferation was inhibited by silencing RPN2 which regulating the glycosylation of EGFR.

## RESULTS

### Association of RPN2 protein expression with CRC clinicopathological features

**Figure 1:**
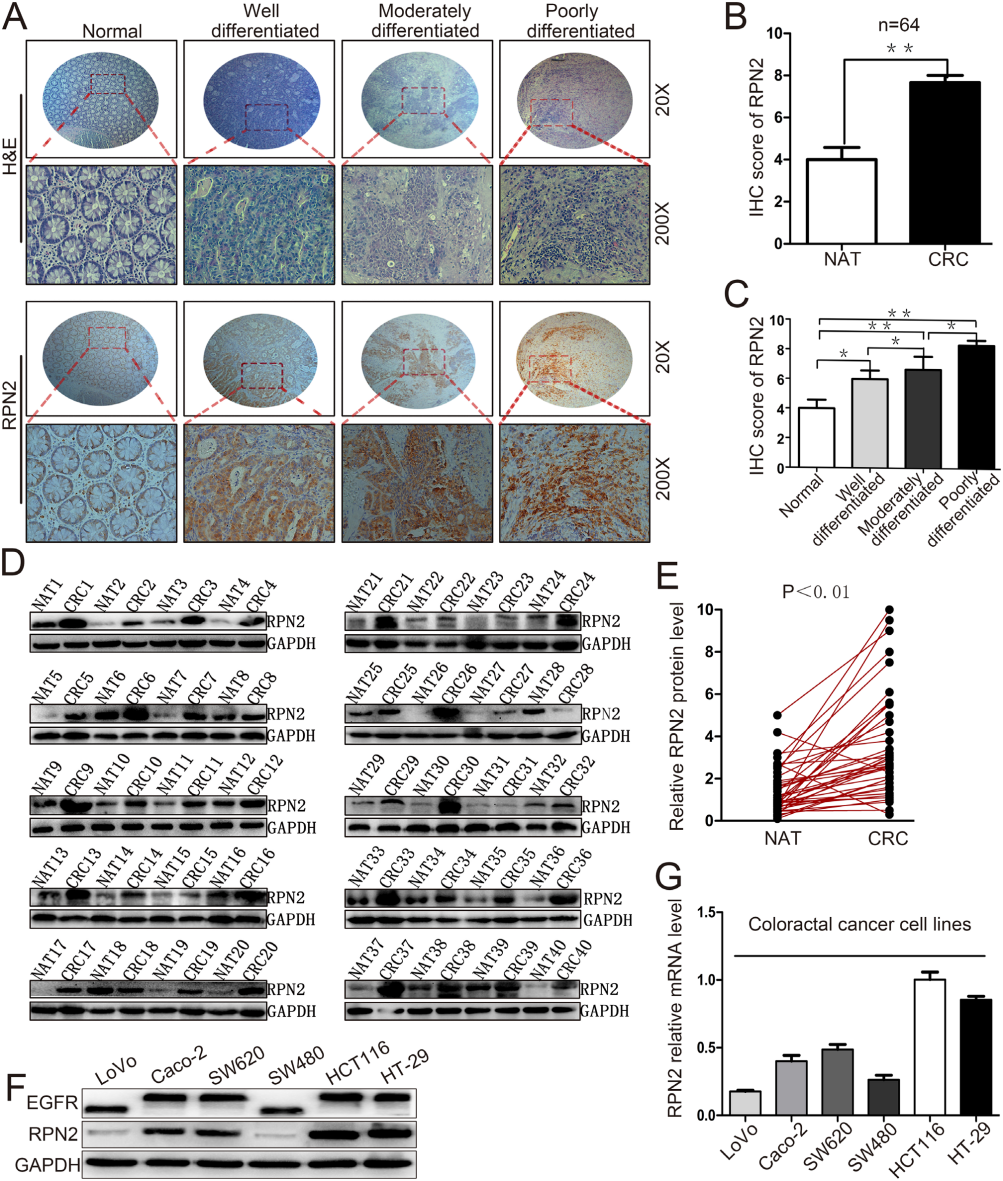
Upregulation of RPN2 is related to tumor growth in colorectal cancer **(A)** Hematoxylin and eosin (H&E) staining and IHC staining of RPN2 protein in normal, well differentiated, moderately differentiated, and poorly differentiated CRC tissues. Microscope images were taken at either ×20 or ×200 magnifications. **(B)** Total IHC score of RPN2 in NATs and CRC tissues (n=64); ^**^, p<0.01; compared with NAT control. **(C)** IHC score of RPN2 in normal, well differentiated, moderately differentiated, and poorly differentiated CRC tissues. ^**^, p<0.01; ^*^, p<0.05 compared with normal control. **(D)** Western blot analysis of RPN2 from NATs and CRC tissues (n=40), GAPDH was used as a loading control. **(E)** RPN2 protein expression levels by quantization of density of protein bands from Western blot in E in colorectal tumors relative to the NATs (n=40, **, p<0.01). **(F)** Western blot analysis of RPN2 and EGFR in cell lysates from CRC cells including LoVo, Caco-2, SW620, SW480, HCT116, HT-29. GAPDH was used as a loading control. **(G)** Relative mRNA level of RPN2 in CRC cell lines.

To investigate the clinical relevance of RPN2 expression in CRC, we firstly examined RPN2 expression in both CRC tissue samples and matched adjacent normal tissue (NAT) sample from a cohort of 64 CRC patients by immunohistochemistry using specific anti-RPN2 antibody. RPN2 expression level was significantly upregulated in CRC tissues compared with matched NATs, and RPN2 was predominantly localized in the cytoplasm of colorectal epithelial cells (Figure 1A and 1B). We also found that the expression level of RPN2 in CRC negatively correlated with the differentiation state of the cancer cells (Figure 1A and 1C). Furthermore, Western blot analysis confirmed that the expression levels of RPN2 in CRC tissues from 40 patients were markedly higher than in matched NATs (Figure 1D and 1E). The protein and mRNA expression levels in CRC cell lines indicated that RPN2 expressions were higher in HCT116 and HT-29 than in other CRC cell lines (Figure 1F and 1G). These results suggested that RPN2 expression levels were upregulated in CRC.

We further investigated whether RPN2 expression level was associated with any clinicopathological variables in 64 CRC specimens, which were classified into 2 groups based on RPN2 IHC staining level: a positive RPN2 expression group (n=28,43.8%) and a negative RPN2 expression group (n=36,56.2%) (Supplementary Figure 1). The results revealed that RPN2 expression level was strongly correlated with several variables including stages (P=0.044), differentiation (P=0.007) and tumor size (P=0.009, mean=40mm), but not with gender, age, tumor location or metastasis (Table 1). These data suggested that RPN2 has a potential role in CRC progression by enhancing cell growth and inhibiting cell differentiation.

**Table 1:** Clinicopathologic characteristics of RPN2 expression in CRC patients

Characteristic	All(n=64)	RPN2			
		Positive(%)	Negative(%)	X^2^	P-value
Gender				0.068	0.795
Men	40	18(45)	22(55)		
Women	24	10(41.7)	14(58.3)		
Age(Years)				0.062	0.803
>55	47	21(44.7)	26(55.3)		
≤55	17	7(41.2)	10(58.8)		
Location				0.498	0.481
Colon	38	18(47.4)	20(52.6)		
Rectal	26	10(38.5)	16(61.5)		
Metastasis				3.521	0.061
Lymph nodes	23	7(30.4)	16(69.4)		
Distant	9	6(66.7)	3(33.3)		
Stages				4.063	0.044*
I-II	32	10(31.3)	22(68.7)		
III-IV	32	18(56.3)	14(43.7)		
Differentiation				10.027	0.007**
Well	9	2(22.2)	7(77.8)		
Moderately	34	11(32.4)	23(67.6)		
Poorly	21	15(71.4)	6(28.6)		
Tumor size				6.836	0.009**
>40mm	25	16(64)	9(36)		
≤40mm	39	12(30.8)	27(69.2)		

*, p<0.05. **, p<0.01.

### RPN2 promotes CRC cell cycle progression and proliferation *in vitro*

**Figure 2:**
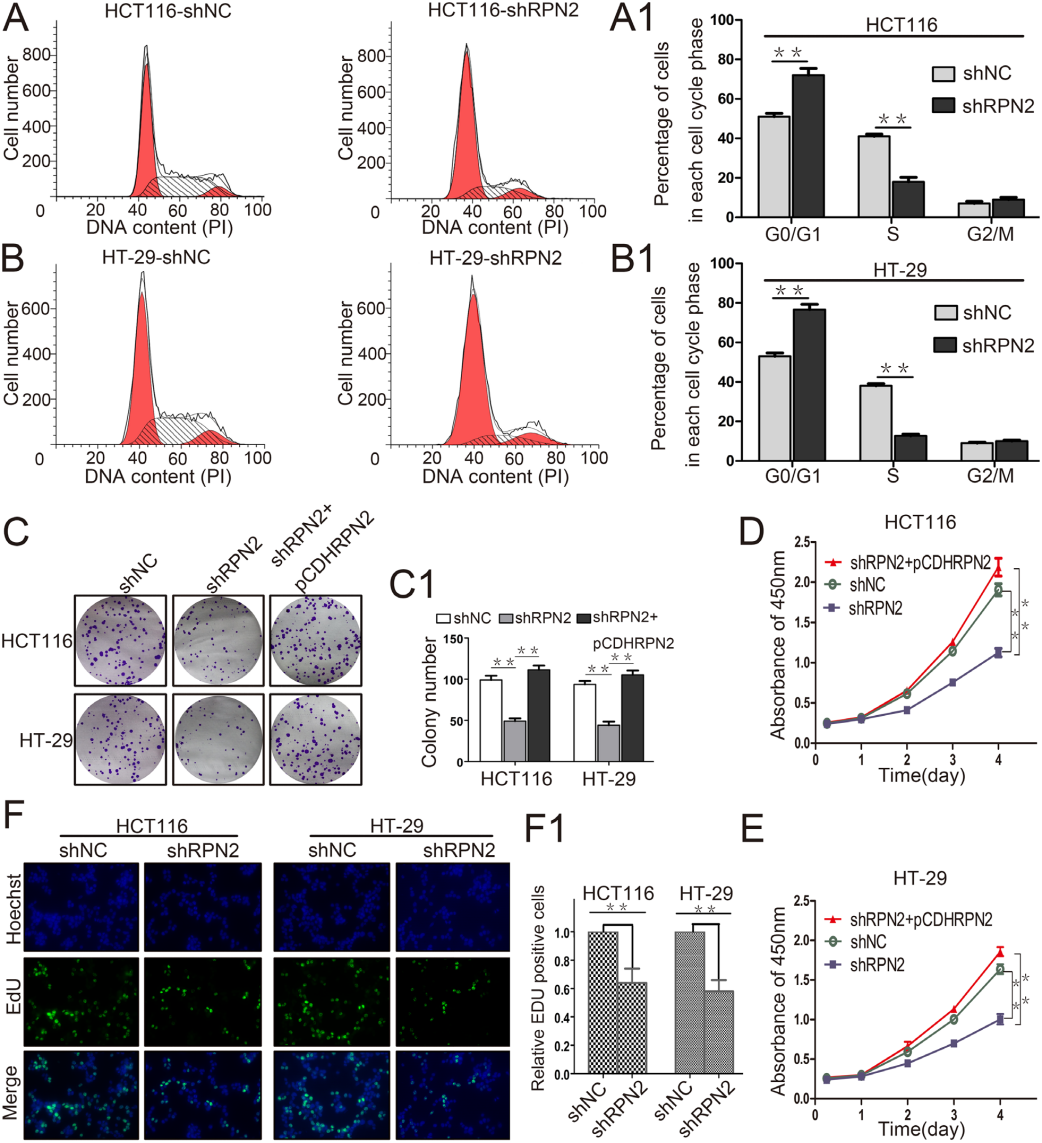
RPN2 knockdown inhibits colorectal cancer cell proliferation and cycle progression *in vitro* **(A** and **A1)** Flow cytometry assays were performed to analyze cell cycle in HCT116-shNC and HCT116-shRPN2 cells. Values at different stages of the cell cycle represent mean±SD from at least three independent experiments. ^**^, p<0.01 compared with control. **(B** and **B1)** Flow cytometry assay were performed to analyze the cell cycle in HT-29-shNC and HT-29-shRPN2 cells. **(C** and **C1)** Colony formation assay of stable HCT116 and HT-29 cells with knockdown of RPN2 (shRPN2), rescue of RPN2 (shRPN2+pCDHRPN2), and negative control (shNC). Values of colony number were shown as mean±SD from at least three independent experiments. ^**^, p<0.01 compared with control. **(D)** CRC cell proliferation were detected by CCK-8 and absorbance at 450 nm at different time-points. Values at the indicated time-points represent mean±SD from at least three independent experiments. ^**^, p<0.01 compared with control. **(E)** CCK-8 assay conducted in HT-29 cells. **(F** and **F1)** The effect of RPN2 silencing on the growth of colorectal HCT116 and HT-29 cancer cells compared with negative control analyzed by EdU proliferation assay. ^**^, p<0.01 compared with control.

To assess the role of RPN2 in CRC cell growth, we examined the effect of RPN2 on cell cycle and proliferation. We established stable clones expressing short hairpin RNA (shRNA) against RPN2 including HCT116-shRPN2 and HT-29-shRPN2, and their respective negative controls, HCT116-shNC and HT-29-shNC. Flow cytometry was used to analyze cell cycle and apoptosis. The results suggested that cells were accumulated mainly at G1 phase in RPN2-depleted cells compared with the negative controls (Figure 2A and 2B), but no change in apoptosis was observed (Supplementary Figure 2A and 2B). To directly observe cell growth, colony formation assays were conducted, and shRPN2 clones exhibited less colonies as compared with the control clones. Meanwhile, the colonies numbers of shRPN2 clones were increased after transfected with lentiviral vector plasmid pCDH-RPN2 which could highly express *RPN2* gene. (Figure 2C). In addition, CCK-8 assay showed that cell growth rate was decreased in stable knockdown of RPN2 cells, and it was restored with RPN2 overexpression (Figure 2D and 2E). These results were further confirmed by EdU staining (Figure 2F). However, negligible impact of RPN2 on cell migration and invasion was seen (Supplementary Figure 2C and 2D). Cells in the G1 phase were decreased in SW480-pCDHRPN2 cells with RPN2 overexpression compared with the controls (Figure 9A and 9B). The results of the EdU staining indicated faster cell growth in SW480-pCDHRPN2 cells than in control cells (Figure 9C and 9D). Combined, these data suggested that RPN2 promoted CRC cell proliferation and RPN2 silencing inhibited cell cycle G1-S phase transition.

### RPN2 silencing inhibits EGFR glycosylation and cell-surface transport

**Figure 3:**
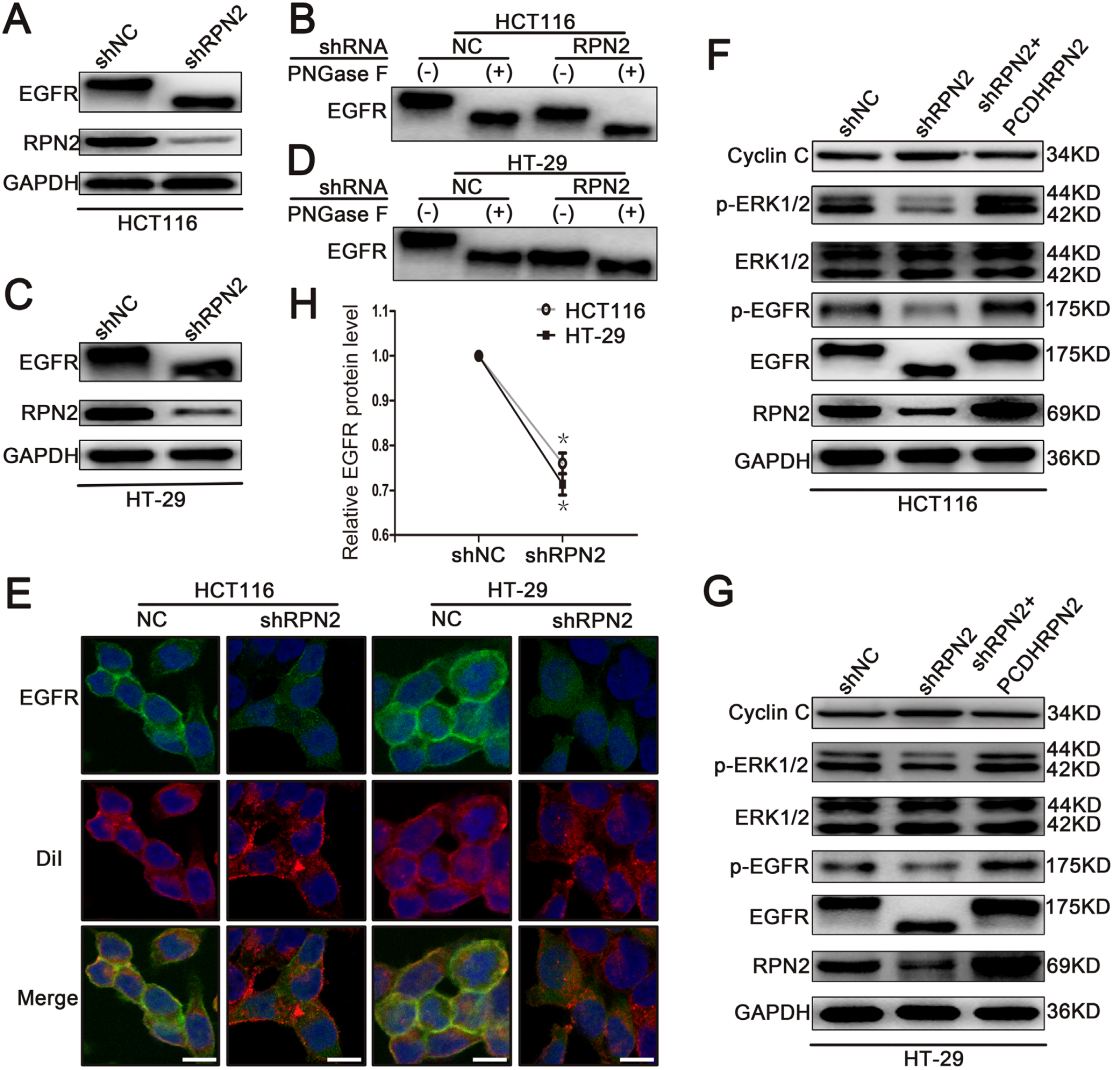
RPN2 silencing regulates glycosylation of EGFR and EGFR/ERK signaling pathway **(A)** Western blot analysis shows the glycosylation status and expression level of EGFR and the silence efficiency of RPN2 in HCT116-shRPN2 cells compared with control. **(B)** The glycosylation status of EGFR is analyzed by Western blot in both HCT116-shRPN2 and HCT116-shNC cells; molecular size shifts are compared to PNGase F digestion treatment. **(C)** Western blot analysis of EGFR and RPN2 in both HT-29-shNC and HT-29-shRPN2 cells. **(D)** Glycosylation status of EGFR analyzed by Western blot in HT-29 cells. **(E)** Localization of EGFR in CRC cells. Confocal microscopy and immunofluorescence of EGFR protein (green) and DiI (red), nuclei are blue (DAPI). Merged images are shown. Scale bar, 10 μm. **(F** and **G)** Relevant proteins of EGFR/ERK signaling pathway analyzed by Western blot in HCT116 and HT-29 cells. **(H)** Relative EGFR protein level in both RPN2 silenced cells and control cells, ^*^, p<0.05 compared with control.

EGFR is a highly glycosylated transmembrane receptor tyrosine kinase (RTK) protein with 11 consensus N-linked glycosylation sites in the extracellular domain. Each glycosylation site contributes approximately 3 kDa to the total molecular weight of this protein. EGFR has been identified as a key driver of proliferation and survival signaling in malignant tumors; therefore, we sought to investigate RPN2 silencing-mediated OST impact on EGFR function. We examined the glycosylation status of EGFR protein using Western blotting in HCT116-shRPN2 and HT-29-shRPN2 cells with 90% RPN2 inhibition, and observed that RPN2 knockdown reduced the total EGFR expression by approximately 25% (Figure 3H) and decreased the molecular weight of EGFR compared to negative control (Figure 3A and 3C). Furthermore, in order to confirm whether the decrease of EGFR molecular weight was actually based on deglycosylation, we treated cell lysate samples with N-glycosidase F (PNGase F) peptide to remove N-glycan chains, which further decreased the molecular weight of EGFR compared to the RPN2-silenced cell lysate samples (Figure 3B and 3D). The PNGase F experiment demonstrated that RPN2-silencing blocked the transfer of most, but not all, N-linked glycans to the EGFR. The molecular weight of EGFR increased in SW480-pCDHRPN2 cells compared with the controls (Figure 9E). These data suggested that RPN2 contributed to the N-glycosylation of EGFR in human CRC cells.

N-linked glycosylation is an important step for the quality control and trafficking of transmembrane glycoproteins such as the EGFR; therefore, we further assessed the RPN2-silencing effects on cell surface EGFR expression in HCT116 and HT-29 cells by immunofluorescence staining. In the negative control group, the results revealed that EGFR was chiefly localized to the plasma membrane, as indicated by DiI staining, which is a lipophilic membrane dye that can gradually spread laterally to the whole cell membrane. However in RPN2-silenced cells, the EGFR was predominantly found in intracellular fraction (Figure 3E), suggesting a change in cellular localization. Meanwhile, the EGFR was mainly found in intracellular fraction in SW480-NC cells with low RPN2 expression, and chiefly observed in the plasma membrane in SW480-pCDHRPN2 cells (Figure 9F). Taken together, these results demonstrated that RPN2-silencing blocks EGFR trafficking to the cell surface.

### RPN2 regulates EGFR/ERK signaling pathway through mediating the glycosylation of EGFR

EGFR is involved in cell proliferation and signal transduction including EGFR/ERK pathway. Mutation of the EGFR kinase domain (KD) increases EGFR tyrosine kinase activity, drives tumorigenesis, and results in tumors that are dependent on RTK signaling for proliferation [28]. Since our data thus far has shown that RPN2 silencing decreased EGFR glycosylation and localization, the effect of RPN2 silencing on EGFR-dependent signaling pathway was also tested. Phosphorylation of EGFR (p-EGFR: Y1068) and ERK1/2 (p-ERK1: T202/Y204, p-ERK2: T185/Y187) were down-regulated in HCT116-shRPN2 and HT-29-shRPN2 cells, without impacting total ERK1/2 expression level. To confirm this phenomenon, we rescued RPN2 expression level in RPN2-silenced cells and found the total EGFR, p-EGFR or p-ERK1/2 expression level were slightly elevated compared with RPN2-silenced cells (Figure 3F and 3G). Cyclin C protein accumulates predominantly in G1 phase cells. Cyclin C protein expression increased in RPN2 low expression cells compared with RPN2 high expression cells (Figure 3F and 3G). Meanwhile, the EGFR/ERK signaling pathway were upregulated in SW480-pCDHRPN2 cells (Figure 9G). EGFR protein expression levels and molecular weight were lower in CRC cell lines with RPN2 low expression (LoVo and SW480) than in CRC cell lines with RPN2 high expression (HCT116 and HT-29) (Figure 1F). These results provided evidence that inhibition of EGFR N-linked glycosylation by RPN2 silencing downregulates the EGFR/ERK signaling pathway.

### Decreased EGFR expression inhibits CRC cell proliferation

The relevance both EGFR and cancer cell malignancy has previously been reported. As shown in Figure 3, RPN2 silencing led to abnormal distribution of EGFR, and reduced EGFR expression level, indicating that EGFR functions were impeded by RPN2 silencing. Since RPN2 could regulate CRC cell cycle progression and proliferation (Figure 2), we further confirmed whether EGFR contributed to CRC cell cycle progression and proliferation.

**Figure 4:**
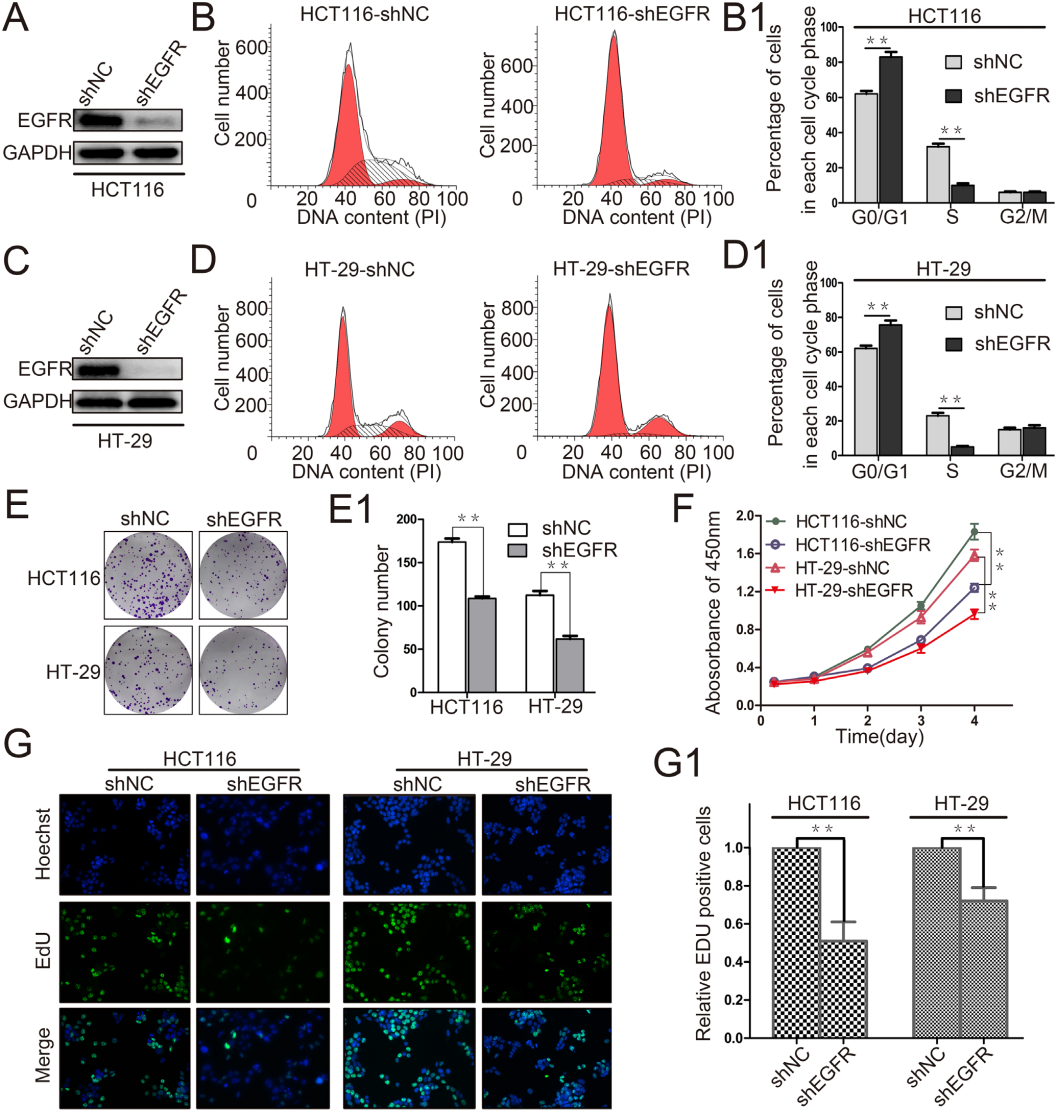
EGFR silencing inhibits colorectal cancer cell proliferation **(A** and **C)** The interference efficiency of EGFR was verified by Western blot in HCT116 and HT-29 cells. **(B, B1, D** and **D1)** Flow cytometry assays were performed to analyze the cell cycle in HCT116 and HT-29 cells. Values at different stages of cell cycle represent mean±SD from at least three independent experiments. ^**^, p<0.01 compared with control. **(E** and **E1)** Colony formation assay of stable HCT116 and HT-29 cells with knockdown of EGFR (shEGFR) or negative control (shNC). Values of colony number were shown as mean±SD from three independent experiments. ^**^, p<0.01 compared with control. **(F)** CRC cell proliferation was detected by CCK-8 and absorbance at 450 nm at different time-points is shown. Values at the indicated time-points represent mean±SD from three independent experiments. ^**^, p<0.01 compared with control. **(G** and **G1)** The effect of EGFR silencing on the growth of colorectal HCT116 and HT-29 cancer cells compared with negative control analyzed by EdU proliferation assay. ^**^, p<0.01 compared with control.

We firstly knocked down EGFR expression in HCT116 and HT-29 cells, and the interference efficiency was checked on Western blotting (Figure 4A and 4C). We found that cell cycle G1-S phase transition was also blocked by EGFR silencing (Figure 4B and 4D). In addition, cell proliferations were analyzed by CCK-8 assay, colony formation, and EdU staining, and results indicated slower cell growth in EGFR-silenced cells than in negative control cells (Figure 4E, 4F and 4G). Cell proliferation slightly decreased in both EGFR- and RPN2-silenced cells compared with EGFR-silenced cells (Supplementary Figure 4A–4D). Taken together, our result demonstrated that EGFR silencing inhibited CRC cell growth.

### Decreased glycosylation of EGFR inhibits the proliferation of CRC cells

Tunicamycin (Tn) is a N-linked glycosylation inhibitor that inhibits protein glycosylation by blocking the first step in the biosynthesis of N-linked oligosaccharides in the endoplasmic reticulum and Golgi. To confirm glycosylation on EGFR mediated by RPN2 play a critical effect on CRC cell growth, we carried out the tunicamycin experiment. We examined the glycosylation status of EGFR protein using Western blotting in HCT116 and HT-29 cells with tunicamycin treatment, and observed that tunicamycin reduced the total EGFR expression and decreased the molecular weight of EGFR compared to control (Figure 8A–8D). MG-132 is an effective protease inhibitor that blocks the ubiquitin-proteasome pathway, we found that the expression of EGFR increased in shRPN2 or tunicamycin treated cells with MG-132 than in control cell (not treated with MG-132) (Figure 8A–8D). Flow cytometry was used to analyze cell cycle. The results suggested that cells in the G1 phase were increased in HCT116-Tn or HT-29-Tn cells compared with the controls (Figure 8F–8I). In addition, cell proliferations were analyzed by CCK-8 assay and EdU staining, and results indicated slower cell growth in tunicamycin treated cells than in control cells (Figure 8E, 8J and 8K). Taken together, our result showed that the glycosylation of EGFR can regulate the proliferation of CRC cells.

### RPN2 promotes xenograft tumor growth, at least in part, through regulating EGFR glycosylation

**Figure 5:**
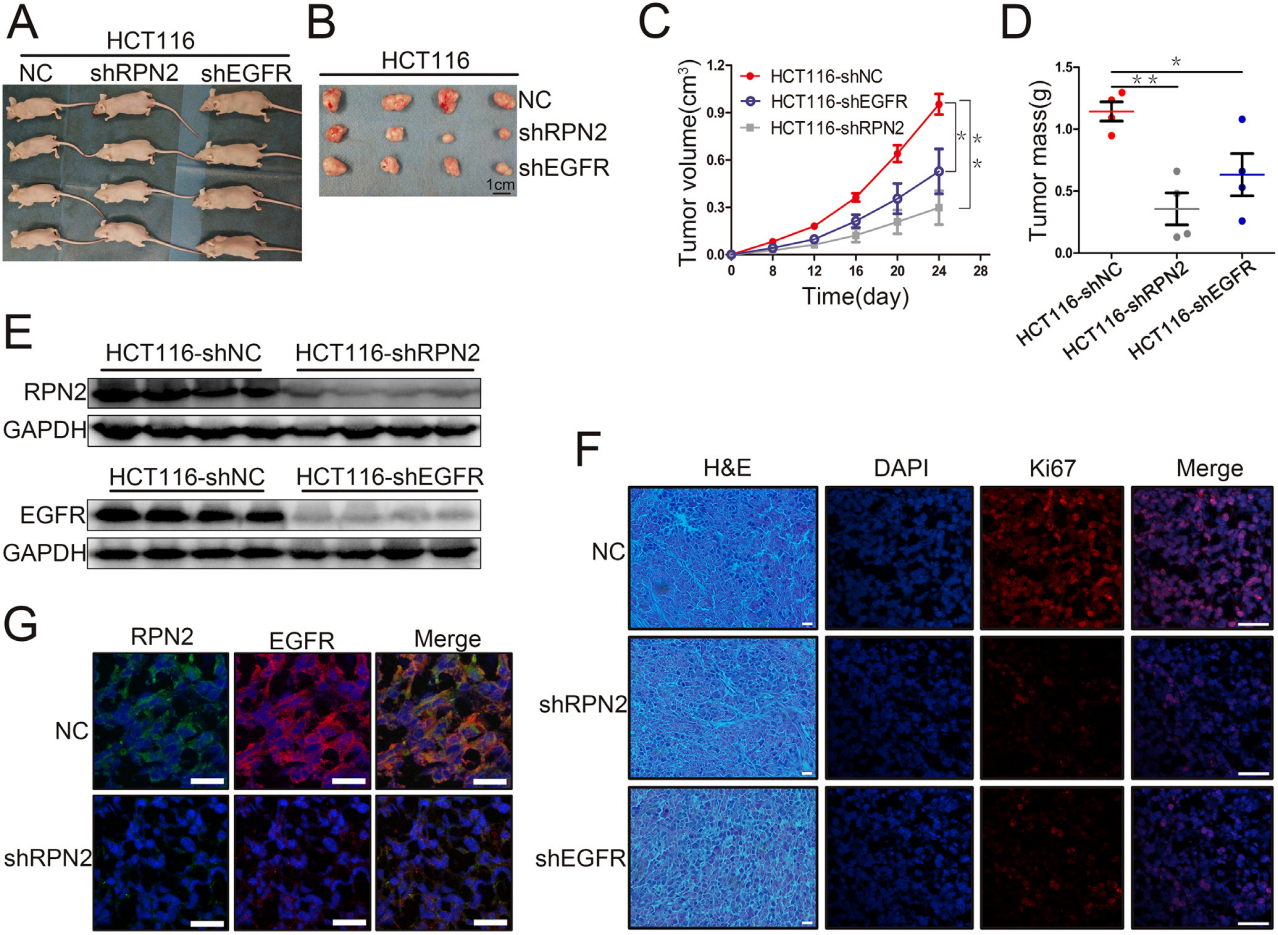
RPN2 or EGFR knockdown suppressed xenograft tumors growth in nude mice **(A)** Growth of tumors in nude mice from RPN2-knockdown, EGFR-knockdown, and control HCT116 cells (n=12). **(B)** Tumor tissues derived from xenograft tumors in nude mice 24 days after inoculation. Scale bar, 1 cm. **(C)** The mean volume of xenograft tumors from HCT116-shRPN2, HCT116-shEGFR, and control HCT116 cells. ^*^, p<0.05. ^**^, p<0.01. **(D)** The mean tumor weight from HCT116-shRPN2, HCT116-shEGFR, and control HCT116 cells. ^*^, p<0.05. ^**^, p<0.01. **(E)** Xenograft tumors tissue protein extracted from HCT116-shRPN2, HCT116-shEGFR, and control HCT116 cells then immunoblot for RPN2 and EGFR. GAPDH was used as a loading control. **(F)** Immunofluorescent staining of xenograft tumor tissues from HCT116-shRPN2, HCT116-shEGFR, and control HCT116 cells for Ki67 (red). Nuclei are blue (DAPI). Merged images are shown. Scale bar, 30 μm. **(G)** Localization of EGFR in tumors of HCT116 in mice. Immunofluorescence staining of RPN2 (green) and EGFR (red) are shown. Nuclei are blue (DAPI). Merged images are also shown. Scale bar, 20 μm.

To extend our *in vitro* findings and to verify that RPN2 had a growth-promoting effect on CRC cells, a xenograft tumor model was established in nude mice. Subcutaneous tumor development of RPN2 or EGFR shRNA-mediated stable knockdown or negative control of HCT116 cells were monitored by measuring the tumor size and weight every 4 days. We found that tumor cells from shRPN2 (P=0.002) or shEGFR (P=0.034) transfections grew more slowly than the negative control in mice (Figure 5A and 5B). Tumor volume and weight in shRPN2- or shEGFR-inoculated mice were significantly decreased compared with negative control mice (Figure 5C and 5D). However, tumor volume and weight were smaller in shRPN2-inoculated mice than in shEGFR-inoculated mice. These results indicated that RPN2 or EGFR silencing suppressed proliferation of CRC cells *in vivo*. In order to further confirm whether these phenomena were caused by RPN2 or EGFR silencing *in vivo*, we extracted the protein from the tumors, and found that the interference efficiency of RPN2 or EGFR was at least 90% *via* Western blotting (Figure 5E). In addition, Ki67 staining was performed to investigate the proliferation activity of tumor tissue with RPN2 or EGFR silencing, and our results revealed that the expression level of Ki67 was higher in control mice than in mice inoculated with HCT116-shRPN2 and HCT116-shEGFR (Figure 5F). Furthermore, we investigated whether RPN2 could regulate EGFR glycosylation in xenograft tumor tissues, and immunofluorescence staining showed that EGFR localization was altered and protein expression decreased by RPN2 silencing (Figure 5G). Taken together, these results indicated that RPN2 silencing suppressed proliferation of CRC cells *in vivo,* at least in part through regulating EGFR glycosylation to alter its localization and expression level.

### RPN2 and EGFR are associated with cell growth in human CRC

**Figure 6:**
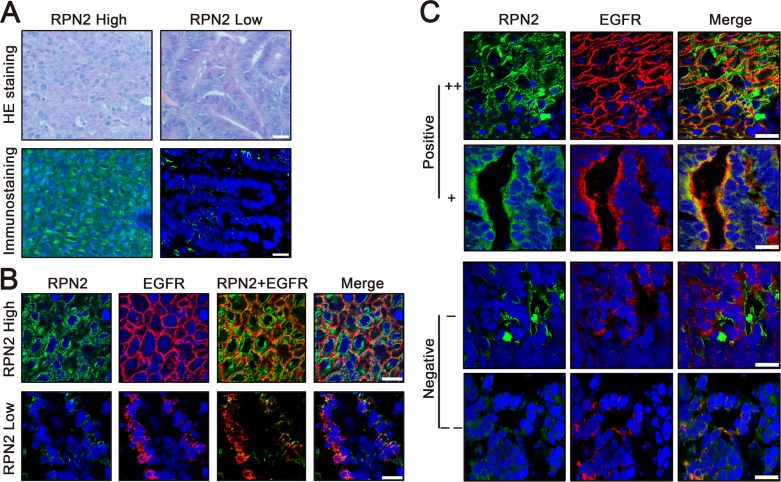
Status of RPN2 and EGFR in human colorectal cancer tissues **(A)** Expression of RPN2 in human CRC tissues. H&E staining and RPN2 immunofluorescent staining (green) of tissue sections were shown. Nuclei are blue (DAPI). Scale bar, 50 μm. **(B)** Localization of EGFR in human CRC tissues with RPN2 high expression and RPN2 low expression. Immunofluorescence staining of RPN2 (green) and EGFR (red) are shown. Nuclei are blue (DAPI). Merged images are also shown. Scale bar, 20 μm. **(C)** The relationship between RPN2 and EGFR in human CRC tissues. Immunofluorescence staining of RPN2 (green) and EGFR (red) are shown. Nuclei are blue (DAPI). Merged images are also shown. According to the expression status of RPN2 and EGFR were divided into positive (++ and +) and negative (− and --) grades, grade ++ and - represents strong staining, grade + and -- represents weak staining. Scale bar, 20 μm.

Immunofluorescence staining suggested that EGFR was mainly distributed in the cell membrane in negative control cells, whereas the intensity of membrane EGFR and total EGFR expression level were downregulated in RPN2-silenced cells (Figures 3 and 5). To further determine whether the expression of RPN2 and EGFR were correlated in CRC, we conducted immunostaining analysis of RPN2 and EGFR in human CRC tissues with RPN2 high expression and RPN2 low expression (Figure 6A). The result demonstrated that EGFR was chiefly localized to the cell membrane in CRC tissues with high RPN2 expression; however, in CRC tissues with low RPN2 expression, EGFR was mainly distributed in the cytoplasm (Figure 6B).

Moreover, to examine the relationship between RPN2 and EGFR in clinical samples, we performed immunofluorescent staining of RPN2 and EGFR in 40 CRC tissues. According to the expression status of RPN2 and EGFR, samples were divided into positive (++ and +) and negative (− and --) grades, with grade ++ and - representing strong staining, grade +, and -- representing weak staining (Figure 6C). The analysis data from the chi-square test revealed that EGFR expression status was significantly associated with RPN2 expression (P<0.01). Pearson correlation coefficient between RPN2 and EGFR was 0.56, which was indicative for high relevance (Table 2). At the same time, the correlation between EGFR expression level and tumor size (mean=39mm, n=40) was analyzed by the chi-square test (P=0.02), the result indicated that the expression of EGFR was significantly correlated with CRC cell growth (Table 3). Taken together, these results suggested that RPN2 regulated the distribution and expression of EGFR to reduce the growth of CRC cells.

**Table 2:** The association between RPN2 and EGFR in colorectal cancer

EGFR	RPN2		Total(%)	X^2^	P-value	Pearson's R
	Positive	Negative				
Positive	16	3	19(47.5)	12.50	<0.01	0.56
Negative	6	15	21(52.5)			
Total(%)	22(55.0)	18(45.0)	40			

**Table 3:** Correlation analysis of EGFR expression and tumor size of CRC

Tumor size	EGFR		Total(%)	X^2^	P-value
	Positive	Negative			
>39mm	15	9	24(60.0)	5.41	0.02
≤39mm	4	12	16(40.0)		
Total(%)	19(47.5)	21(52.5)	40		

## DISCUSSION

Various reports have found that RPN2 is associated with the growth of cancer cells [7–10]; however, the specific development mechanisms have not been clearly elucidated. Recently, RPN2 protein immunostaining exhibited a significant association with poor prognosis in CRC patients [13]. In this study, we found a vital role of RPN2 in CRC progression and identified the possible involvement of a EGFR-mediated mechanism. This finding revealed that RPN2 promoted CRC cell proliferation through mediating EGFR glycosylation, as shown in CRC cells, xenografted tumors in mice, and human CRC tissues. Moreover, clinicopathological analysis revealed that RPN2 expression level was strongly correlated with several variables including disease stage, differentiation, and tumor size. We found that RPN2 protein levels were markedly upregulated in primary CRC tissues compared with adjacent non-tumor tissues. In addition, silencing of RPN2 decreased the glycosylation of EGFR and reduced its membrane localization and expression level. We also found that the expression of EGFR in CRC tissues was positively correlated with the expression level of RPN2 and tumor size. Taken together, we speculate that RPN2 is crucial for CRC cell proliferation and acts as a modulator of glycosylation status of EGFR, which is related to the localization and expression of EGFR.

N-linked glycosylation is a process in which a N-glycan is linked to a free-NH2 group of a specific asparagine (N-X-S/T, X!=P) in a nascent peptide chain and is a protein modification critical for glycoprotein folding, stability, and cellular localization [29]. Protein glycan play crucial roles in various biological processes including cell adhesion, proliferation, and cellular signaling [30]. Furthermore, N-glycan number and branching cooperate to regulate glycoprotein levels and cell proliferation and differentiation [31]. In eukaryotic cells, glycosylation has significant effects on protein folding, conformation, distribution, stability, and activity, while dysfunction of protein glycosylation may likely lead to development of cancer [32]. Taken together, we estimate that N-linked glycosylation can regulate the proliferation of cancer cells by adjusting glycoprotein expression levels and cellular functions.

Human EGFR has been found to be highly N-glycosylated but not O-glycosylated [33], and there are 11 N-glycosylation sites in the extracellular domain [26]. An increasing number of studies have reported the importance of N-glycosylation on the functional properties of EGFR, including cell surface expression [17, 18], ligand binding [19], dimerization [21], interaction with membranes [22], and endocytosis [23]. These functional properties are intrinsically connected with EGFR conformation. Research has shown that conformational stability of EGFR is influenced partly by N-linked glycosylation [20]. An unstable protein conformation by deglycosylation may weaken the EGFR, thus impacting its functional capacity. For example, in the presence of tunicamycin, an inhibitor of N-linked glycosylation, a 130-135 kDa immature EGFR protein is synthesized which apparently does not reach the cell surface and does not acquire the capacity to bind EGF [24]. EGFR mediates the mitogenic response of cells to EGF and transforming growth factor-α (TGF-α) [34]. It is well known that these ligands (EGF and TGF-α) could activate EGFR/ERK signaling pathway to promote cell proliferation [35]. Previous studies have also shown that EGFR signaling can be modulated by N-glycosylation [36]. To sum up, we posit that the glycosylation status of EGFR may regulate its functional properties and the EGFR/ERK signaling pathway.

**Figure 7:**
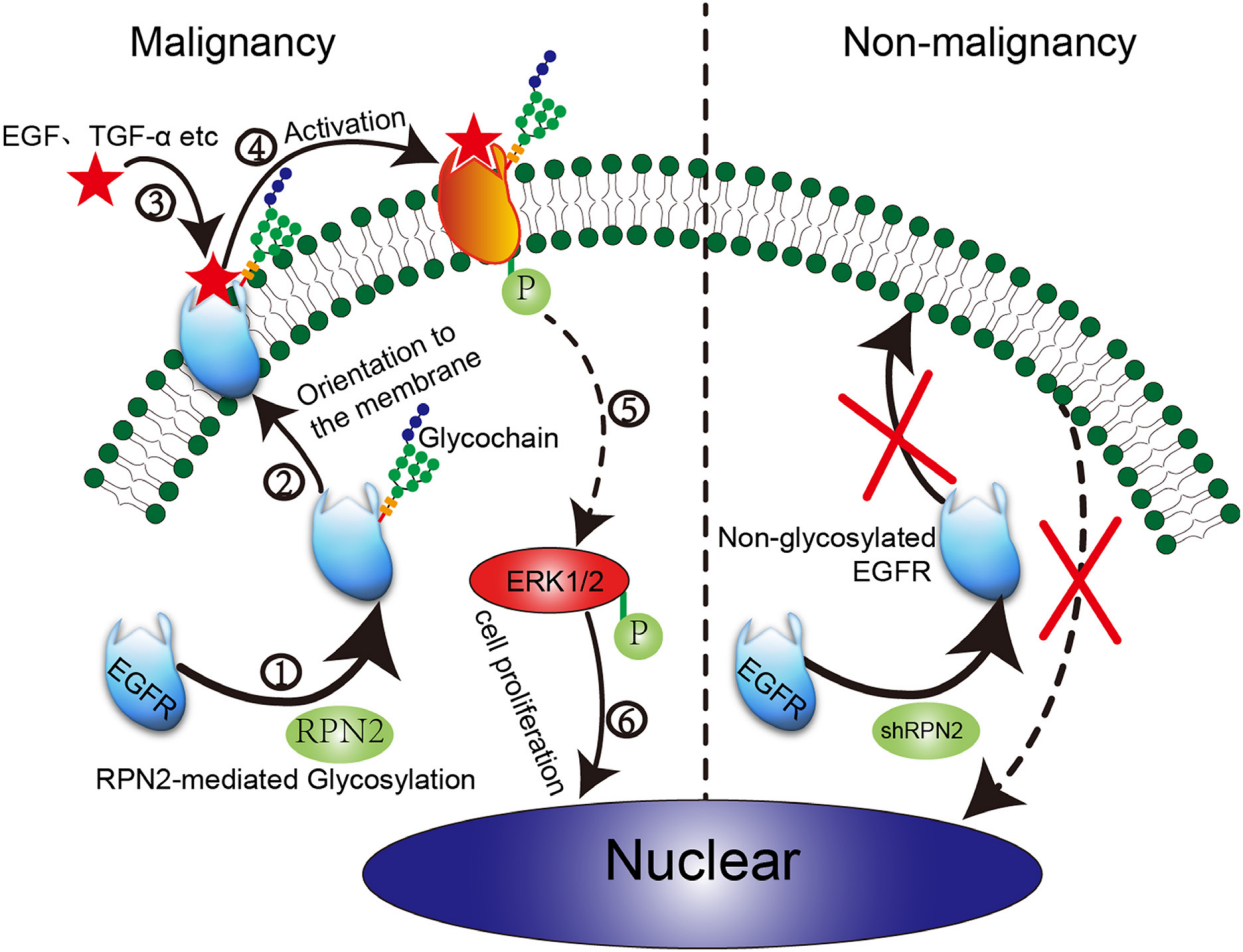
Proposed model of RPN2/EGFR/ERK controlling cell proliferation and cancer progression In colorectal malignant tumors with high RPN2 expression, RPN2 is involved in mediating the glycosylation of EGFR. Glycosylated EGFR is transported to the cell surface through the identification and localization of glycochain. After mutual combination of EGFR and ligands (EGF and TGF-α), glycosylated EGFR is activated to phosphorylated EGFR (p-EGFR), eventually leading to ERK activation, and p-ERK enters the nucleus to promote cell proliferation and malignancy. However, in colorectal non-malignant tumors with low RPN2 expression (shRPN2), RPN2/EGFR/ERK signal is impaired leading to decreased cell growth.

We found that EGFR mRNA levels were not significantly reduced in RPN2-silenced cells (Supplementary Figure 3); therefore, we speculated that RPN2 silencing may decrease the glycosylation of EGFR. The RPN2 protein is a part of an oligosaccharyltransferase (OST) complex that is critical for the development of protein N-glycosylation. Several lines of evidence revealed that OST activity is mediated partly by RPN2 [5]. In addition, OST inhibition causes cell cycle arrest accompanied by induction of status of N-linked glycosylation, cell morphology changes, and all hallmarks of senescence [29]. Therefore, we hypothesized that RPN2 silencing may affect OST activity, resulting in weakening glycosylation of the EGFR, thereby reducing its distribution on the cell surface, and decreasing its total expression levels by promoting its degradation. RPN2 silencing reduced EGFR expression level and cell-surface transport, activation of EFGR, and ultimately decreased the expression of p-ERK1/2 (Figure 7). However, in additional to mediating EGFR glycosylation, RPN2 can physically interact with both Y^216^-phosphorylated and unphosphorylated GSK3β to regulate the malignancy of breast cancer [37]. Admittedly, there is still no direct evidence that RPN2 participates in the transcriptional regulation of EGFR. A previous study found that μ-opioid receptor (MOR) cell surface expression and localization was regulated by its direct interaction with ribophorin I (RPN1) [38] which is also a component of the OST complex [39]. Therefore, both RPN2 and RPN1 can regulate protein localization and expression in different ways.

**Figure 8:**
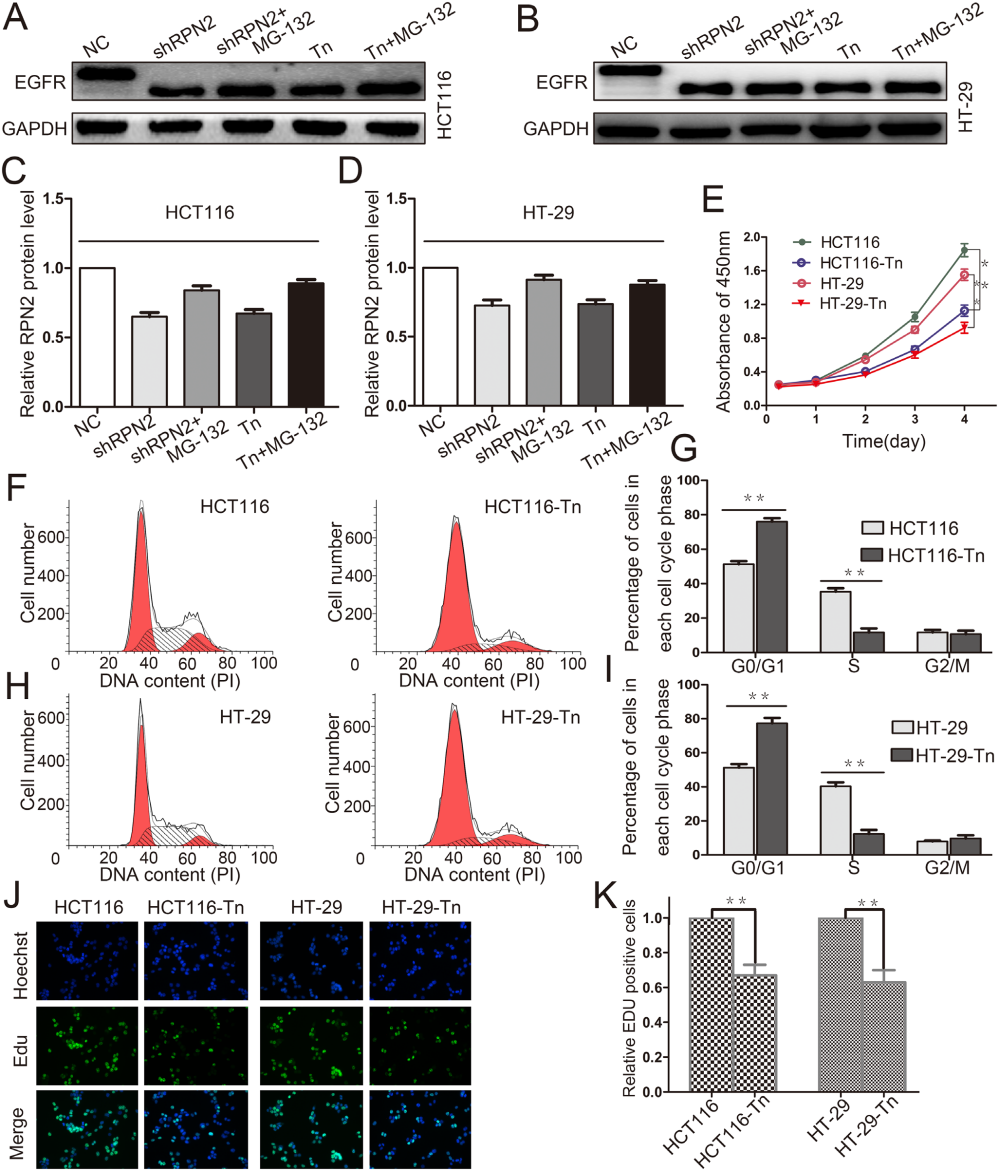
Decreased glycosylation of EGFR inhibits the proliferation of CRC cells **(A, B, C** and **D)** Western blot analysis of EGFR in HCT116 and HT-29 cells with different treatments including shRPN2, shRPN2 and MG-132, Tn, Tn and MG-132, and control, GAPDH was used as a loading control. **(E)** CRC cell proliferation were detected by CCK-8 and absorbance at 450 nm at different time-points. Values at the indicated time-points represent mean±SD from at least three independent experiments. ^**^, p<0.01 compared with control. **(F, G, H** and **I)** Flow cytometry assays were performed to analyze the cell cycle in HCT116 and HT-29 cells. Values at different stages of cell cycle represent mean±SD from at least three independent experiments. ^**^, p<0.01 compared with control. **(J** and **K)** The effect of EGFR glycosylation on the growth of colorectal HCT116 and HT-29 cancer cells compared with control analyzed by EdU proliferation assay. ^**^, p<0.01 compared with control.

**Figure 9:**
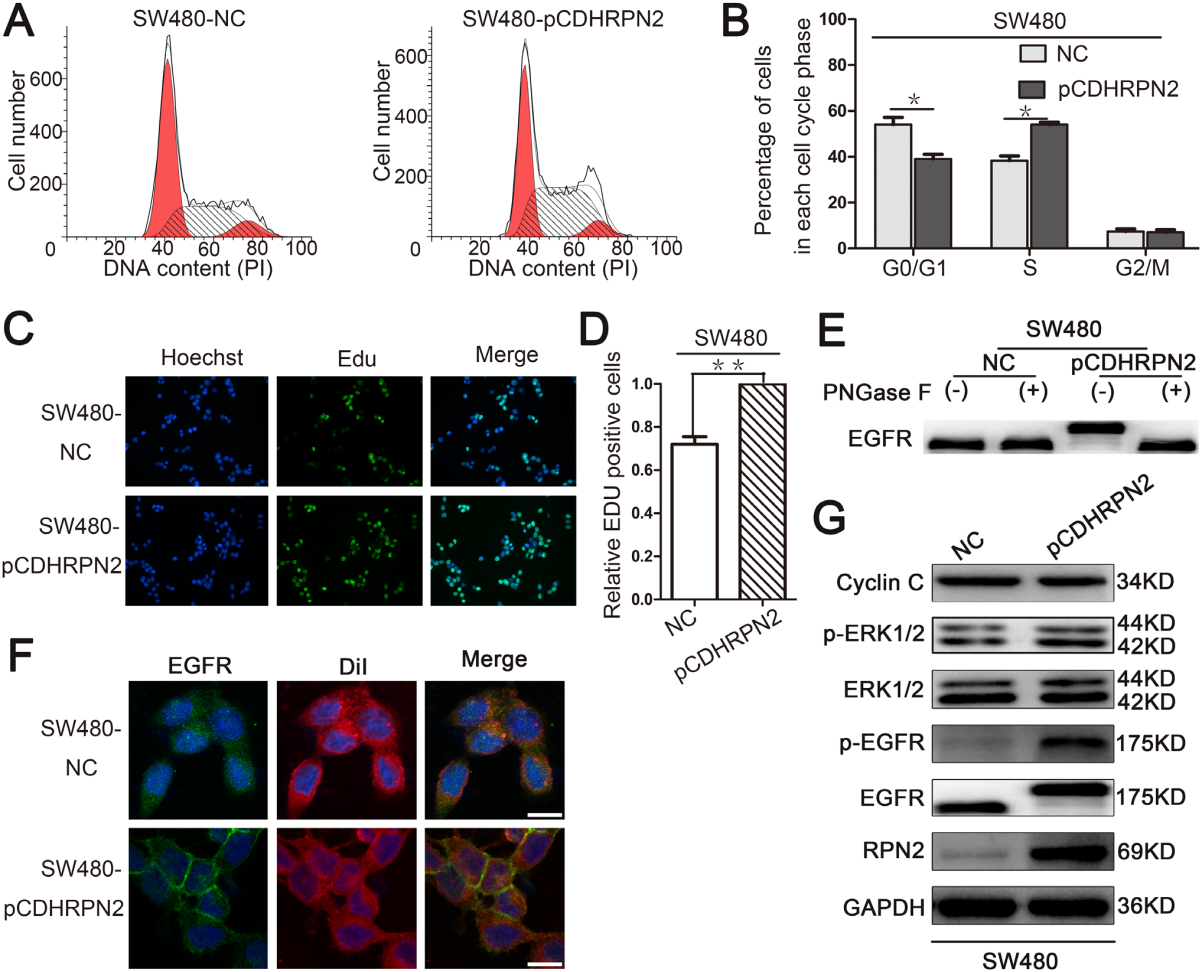
RPN2 overexpression promotes cancer cell proliferation by modulating the glycosylation of EGFR **(A** and **B)** Flow cytometry assays were performed to analyze the cell cycle in SW480 cells. Values at different stages of cell cycle represent mean±SD from at least three independent experiments. ^*^, p<0.05 compared with control. (**C** and **D**) The effect of RPN2 overexpression on the growth of colorectal SW480 cancer cells compared with control analyzed by EdU proliferation assay. ^**^, p<0.01 compared with control. (**E**) Glycosylation status of EGFR analyzed by Western blot in SW480 cells. (**F**) Localization of EGFR in CRC cells. Confocal microscopy and immunofluorescence of EGFR protein (green) and DiI (red), nuclei are blue (DAPI). Merged images are shown. Scale bar, 10 μm. (**G**) Relevant proteins of EGFR/ERK signaling pathway analyzed by Western blot in SW480-NC and SW480-pCDHRPN2 cells.

In the xenograft tumor model study, we found that tumor volume and weight in mice inoculated with RPN2- or EGFR-silenced CRC cells were significantly decreased compared with control mice. However, tumor volume and weight were smaller in mice with RPN2-silenced than in EGFR-silenced mice. Therefore, we concluded that RPN2 silencing suppressed proliferation of CRC cells *in vivo* at least in part through regulating EGFR glycosylation. In addition, we examined the expression of some genes known to be critical for cell cycle G1-S phase transition in HCT116-shRPN2 and HCT116-shNC cells (Supplementary Figure 3). We found the mRNA level of CDK1 significantly decreased in RPN2-silenced cells. CDK1 plays important roles in the regulation of the G2-M transition; however, CDK1 is also capable of regulating G1 progress and G1-S transition [40, 41]. It was previously reported that RPN2-knockdown promoted GSK3β-mediated suppression of heat shock proteins (HSP) in human breast cancer cells [37]. In addition, HSP inhibition downregulated CDK1 expression in human breast cancer cells [42]. Consequently, we speculate that RPN2 silencing may reduce CDK1 expression *via* promoting GSK3β-mediated suppression of heat shock proteins in CRC.

RPN2 is an important molecular marker in various cancers and has been associated with drug resistance in solid cancers, including breast cancer [12], oesophageal squamous cell carcinoma [43], non-small-cell lung cancer [8], and gastric cancer [44]. RPN2 is also associated with the migration and invasion in cancers, including breast cancer [36], laryngeal squamous cell carcinoma [45], and gastric cancer [46]. Moreover, various studies have shown that cancer cell proliferation is inhibited by RPN2 silencing [7–10]. In various human malignant tumors, RPN2 silencing was correlated with reduced tumor growth and distant metastasis and increased sensitivity to chemotherapy drugs response. Therefore, RPN2 may serve as a therapeutic target and prognostic biomarker for CRC; however, validation studies are still needed.

In conclusion, this study provides evidence that N-glycosylation of EGFR can regulate CRC malignancy. Our data demonstrate RPN2-mediated glycosylation of EGFR regulates CRC cell proliferation through affecting the G1-S transition. Further studies are needed to further address the role of RPN2 and whether there are other mechanisms and functions in CRC.

## MATERIALS AND METHODS

CRC cells were obtained from the tumor laboratory of the First Affiliated Hospital of Chongqing Medical University, China. Lentivirus plasmid LV3 (H1/GFP&Puro)-RPN2 (human), LV3 (H1/GFP&Puro)-EGFR (human) and pCDH-CMV-MCS-EF1-CopGFP-T2A-puro were purchased from GenePharma company. The following antibodies were purchased from Abcam (Cambridge, MA): mouse anti-RPN2 (ab156701), rabbit anti-EGFR (ab52894), rabbit anti-EGFR (phosphate Y1068, ab32430), rabbit anti-ERK1 (pT2002/pY204) + ERK2 (pT185/pY187) (ab76299), rabbit anti-Ki67 (ab92742). Rabbit anti-GAPDH (10494-1-AP), rabbit anti-cyclin C (26464-1-AP) and mouse anti-ERK1/2 (66192-1-Ig) were purchased from Proteintech Group, Inc. (Proteintech, China). Alexa Flour 488-labeled goat anti-mouse IgG, Alexa Flour 488-labeled goat anti-rabbit IgG, Alexa Flour 555-labeled donkey anti-rabbit IgG, MG-132 and DiI were purchased from Beyotime, Inc. (Beyotime, China). Tunicamycin (Sigma-Aldrich, USA).

### Clinical samples and immunohistochemical staining

The total of 64 pairs of CRC tissue samples and matched adjacent normal tissues were derived from patients with surgical procedures at the First Affiliated Hospital of Chongqing Medical University, China. All patients in the study provided written consent, and were approved by the Ethics Committee from the First Affiliated Hospital of Chongqing Medical University. Patients with hereditary syndromes, e.g. Familial adenomatous polyposis (FAP), Lynch syndrome or hereditary nonpolyposis colorectal cancer (HNPCC), or inflammatory syndromes were pre-screened and excluded from this study. The preoperational chemoradiotherapy or chemotherapy could significantly influence the expression of biomarkers. Hence none of the patients used in this study received treatment prior to surgery. Immunohistochemical staining was performed using paraffin-embedded sections of biopsies from CRC patients and controls, all tissues were fixed with 10% paraformaldehyde. Paraffin sections were placed in incubators kept at 55°C for 4 hours and then immersed in two consecutive washings in xylol for 20 min to remove paraffin. Sections were hydrated with different concentrations of ethanol including 100%, 95%, 85%, 70% and deionized water respectively, sections were then immersed in citrate buffer solution (0.01 mol/L, pH 6.0) and heated to repair antigen. 0.5% Triton-x-100 was incubated 30 min after washing in PBS. Biotin-streptavidin HRP detection systems (ZSGB, China) were then used to stain the section according to the manufacturer's instructions. These sections were incubated with a primary antibody targeting RPN2 (1:100) at 4°C for overnight. The presence of brown chromogen in the cytoplasm of target cells indicated positive immunoreactivity. The quality of immunostaining was ensured by a negative control incubated without the primary antibody.

### Cell culture, transfections, cell clone, and infection

HCT116 and HT-29 cell lines were purchased from ATCC and cultured in DMEM (Gibco, USA) with 10% fetal bovine serum (BI, ISR) and incubated at 37°C in a humidified atmosphere containing 5% carbon dioxide. Cell lines stably expressing RPN2 shRNA, EGFR shRNA or control non-target shRNA were established using a vector-based shRNA technique and the Human RPN2 shRNA target 5′-GCCACTTTGAAGAACCCAATC-3′, the Human EGFR shRNA target 5′-GCCACAAAGCAGTGAATTTAT-3′ and control shRNA target 5′-TTCTCCGAACGTGTCACGT-3′. Briefly, each fragment was subcloned into LV3 (GenePharma, China). Recombinant lentiviruses were produced following the manufacturer's instructions. Then lentiviral particles were produced in HEK293T cells, after infection, cell lines were generated by selection with 2 ug/ul puromycin (Invitrogen). For overexpressing RPN2, Human RPN2 editing sequences were amplified by PCR with the following primers, F: GGAATTCATGGCGCCGCCGGGTTCAA, R: TTGCGGCCGCCTAATGTGCTGTTCTCTTG, which were cloned into the lentiviral vector plasmid pCDH-CMV-MCS-EF1-CopGFP-T2A-puro at unique *Eco* RI and *Not* I sites. Each of the plasmids was transfected into the cell line with Lipofectamine 2000 (Invitrogen) according to the manufacturer's instructions.

### Protein extraction and western blot analysis

Tissue and cell of proteins extraction use RIPA cell lysis buffer (p0013B) from Beyotime Biotechnology according to the manufacturer's instructions, phosphorylated protein extraction requires the addition of phosphorylated protease complex inhibitors (Beijing Dingguo, China). Protein samples (30-50 ug) were separated by SDS-PAGE gels, transferred onto PVDF membranes, and blocked with 5% skim milk. Anti-RPN2 (1:2000), anti-EGFR (1:8000), anti-GAPDH (1:4000), anti-pEGFR (1:5000), anti-ERK1/2 (1:2000), and anti-pERK1/2 (1:6000) were used as the primary antibodies which were diluted with 5% BSA and 4°C overnight. Two secondary antibodies (HRP goat anti-mouse and anti-rabbit IgG antibodies) were used at a dilution of 1:6000. Bound antibody was visualized by chemiluminescence using Western Bright ECL HRP substrate (Advansta, USA), and luminescent images were analyzed with an Image Lab.

### RNA extraction, reverse transcription, and quantitative real-time PCR (qRT-PCR)

Total RNA was extracted from cultured cells using Trizol reagent (Invitrogen, USA) according to the manufacturer's protocol. cDNA was synthesized from isolated RNA using PrimeScript RT Reagent Kit with gDNA Eraser (TAKARA, Japan) following the manufacturer's instructions. Quantitative real-time PCR was performed with UtraSYBR Mixture (CWBIO, China). qRT-PCR analysis was conducted using primers for human RPN2 (forward: 5′-GCCAGACAACAAGAACGTGT-3′; reverse: 5′-GACCACATCAGCCACATTCC-3′). β-actin (forward: 5′-ATTGCCGACAGGATGCAGA-3′; reverse: 5′-GAGTACTTGCGCTCAGGAGGA-3′) was used for normalization, The relative amounts of gene expression were measured using the 2(−Delta Delta C(T)) method. The reactions were performed using a real-time PCR system. The other primer sequences used for qRT-PCR are listed in Additional file 4: Supplementary Table 1. All reactions were performed in triplicate.

### Cell cycle analysis, CCK-8 assay, EdU incorporation assay, colony formation assay

Cell cycle and apoptosis were analyzed by flow cytometry from Academy of Life Sciences (Chongqing Medical University, China). For cell cycle analysis, 1×10^6^ cells were harvested and fixed at 4°C overnight with 75% ethanol.

An Enhanced Cell Counting Kit-8 (CCK-8) (Beyotime) was used in the cell viability assay, wherein 2×10^3^ cells/well was seeded in 96-well plates. Next, 10 ul of CCK-8 solution was added to each well after 6 h, 24 h, 48 h, 72 h and 96 h incubation. The plate was further incubated for 2 h at 37°C. The absorbance at 450 nm was measured using a microplate reader.

To further assess proliferation, RPN2- and EGFR-silenced cells were seeded in 96-well plates and incubated under standard conditions in complete media. Cell proliferation was determined by incorporation of 5-ethynyl-2′-deoxyuridine (EdU) using an EdU Cell Proliferation Assay Kit (Ribobio, China). The cell nuclei were stained with 4′,6-diamidino-2-phenylindole (DAPI) (Beyotime, China) at a concentration of 1 ug/ml for 8 minutes. The proportion of cells incorporating EdU was determined by fluorescence microscopy.

For colony formation assay, the cells were seeded at a density of 500 cells/well in 6-well plates. After 10 days, the cells were fixed in fresh paraformaldehyde and stained with a crystal violet cell colony staining kit (GenMed, USA). Colony formation was counted with Adobe Photoshop software.

### Migration and invasion assays

For trans-well migration assays, cells (1×10^5^) were plated in the top chamber of non-coated membranes (24-well insert; pore size, 8 μm; BD Biosciences, San Jose, USA). For invasion assays, Matrigel (BD Biosciences) was polymerized in trans-well inserts for 45 min at 37°C prior to seeding. In both assays, cells were seeded in the top chamber with medium without serum, the lower chamber was filled with 10% FBS. Cells were incubated for 24 h and the cells remaining in the top chambers or on the upper membrane of the inserts were carefully removed by a cotton swab. The cells that migrated through the membrane and adhered to the lower surface of the membrane were fixed with paraformaldehyde and stained with crystal violet. For quantification, the cells were counted under a microscope in four random fields.

### Analysis of N-linked glycosylation of EGFR

The presence of glycans in EGFR was determined using peptide-N-glycosidase F (PNGase F). For treatment with PNGase F (Takara, Japan), the supernatant was concentrated 12-fold using Amicon tubes with a molecular weight cutoff of 10 kDa (Millipore, USA). Next, an immunoprecipitation assay was conducted. EGFR antibody (4 ug) was added to the supernatant and incubated on a rocking platform for 4 h at 4°C. Protein A + G agarose beads (Beyotime) were added and incubated for an additional 16 h at 4°C. After incubation, the samples were washed five times with cell lysis solution. The purified EGFR was pre-denatured in 0.2M glycoprotein denaturing buffer at 100°C for 3 min, and the denatured proteins were treated with PNGase F in a mixture with stabilizer solution and deionized water at 37°C for 15 h according to the manufacturer's instructions. Finally, the digested proteins were analyzed by immunoblot.

### Immunofluorescence staining

For immunofluorescence, cells were fixed with fresh 4% paraformaldehyde and labeled with a DiI cell membrane red fluorescent probe at 37°C for 15 min. Cells were permeabilized and blocked with 0.5% Triton-X-100 in PBS for 15 min and 4% BSA at 37°C for 1 h, respectively. Subsequently, the cells were incubated with an anti-EGFR (1:400) antibody overnight at 4°C and labeled with Alexa Flour 488-labeled goat anti-rabbit IgG (1:800) secondary antibody at 37°C for 1 h. Nuclei were stained with DAPI. For tissue immunofluorescence, frozen sections were removed from −80°C and thawed at room temperature for 1 h. Cells were fixed with cold methanol at 37°C for 20 min. Cells were permeabilized and blocked with 0.2% Triton-X-100 in PBS for 15 min and 4% BSA at 37°C for 1 h, respectively. After that, samples were incubated with primary antibodies containing aiti-RPN2 (1:150), anti-EGFR (1:400), and Ki67 (1:400) overnight at 4°C and then labeled with Alexa Flour 488-labeled goat anti-mouse IgG and Alexa Flour 555-labeled donkey anti-rabbit IgG secondary antibodies at 37°C for 1 h. The nuclei were counterstained with DAPI. All staining were observed using a confocal microscope.

### Tumor xenografts in nude mice

Male nude mice aged 4 to 5 weeks were purchased from Animal Experimental Center of Chongqing Medical University. All animal care and handling procedures were performed in accordance with study protocols approved by the Ethics Committee of Chongqing Medical University. Animals were maintained under specific pathogen-free conditions at Chongqing Medical University. A total of 6×10^6^ cells in 100 ul of PBS were injected into each flank of nude mice (4 mice/group). Tumor growth situation was monitored by measuring tumor diameters every 4 days. Both maximum (L) and minimum (W) length of the tumor were measured using a slide caliper, and the tumor volume was calculated using the relationship: ^1^/_2_LW^2^. Mice were euthanized after 24 days, and tumors were collected, weighed, and analyzed.

### Statistical analysis

All statistics were analyzed using SPSS19.0 (SPSS Inc. Chicago, IL, USA). The data presented in bar graphs are the mean±SD of at least three independent experiments and evaluated by two-tailed Student′s *t* test. Immunohistochemical (IHC) score was performed as described previously [47]. Correlation between gene expression and distinct clinicopathological characteristic was analyzed by the Chi-square and Fisher's exact test. Statistical significance was set at P<0.05.

## SUPPLEMENTARY TABLE AND FIGURES


