# Supplementary material for: RPN2 promotes colorectal cancer cell proliferation through modulating the glycosylation status of EGFR
Source: Oncotarget. 2017 Aug 7;8(42):72633–51. doi: 10.18632/oncotarget.20005 (PMC5641158; doi:10.18632/oncotarget.20005)
Supplement: Supplementary file 1 [file oncotarget-08-72633-s001.pdf]

RPN2 promotes colorectal cancer cell proliferation through modulating the glycosylation status of EGFR

SUPPLEMENTARY MATERIALS

Supplementary Table 1: Quantitative real-time PCR primer sequences

Name	Primer	Name	Primer
RPN2	F:GCCAGACAACAAGAACGTGT	EGFR	F:TCTACAACCCCACCACGTAC
	R:GACCACATCAGCCACATTCC		R:TCGCACTTCTTACACTTGCG
CDK1	F:ATGAAGTGTGGCCAGAAGTG	CCND1	F:ACAGATCATCCGCAAACACG
	R:TGACATGGGATGCTAGGCTT		R:GGCGGTAGTAGGACAGGAAG
CDK2	F:TCCGGATCTTTCGGACTCTG	CCNE1	F:GGAAGAGGAAGGCAAACGTG
	R:ACAAGCTCCGTCCATCTTCA		R:TTTGTCAGGTGTGGGGATCA
CDK4	F:CTTCCCATCAGCACAGTTCG	ERK1	F:AAGTACATCCACTCCGCCAA
	R:GGGGTGCCTTGTCCAGATAT		R:CATGCTCAGGATCGGCAATC
CDK6	F:CCAGGCAGGCTTTTCATTCA	ERK2	F:CGCTACACCAACCTCTCGTA
	R:AGGTCCTGGAAGTATGGGTG		R:TAGGTCTGGTGCTCAAAGGG
NF-κB	F:CGCATCCAGACCAACAACAA	GADD45A	F:GCTGGTGACGAATCCACATT
	R:GCACAGCATTCAGGTCGTAG		R:TCCATGTAGCGACTTTCCCG
SOCS3	F:GGCCACTCTTCAGCATCTCT	CDC25A	F:CTACTGATGGCAAGCGTGTC
	R:TTAAAGCGGGGCATCGTACT		R:TCTCTCTCACATACCGGCAC

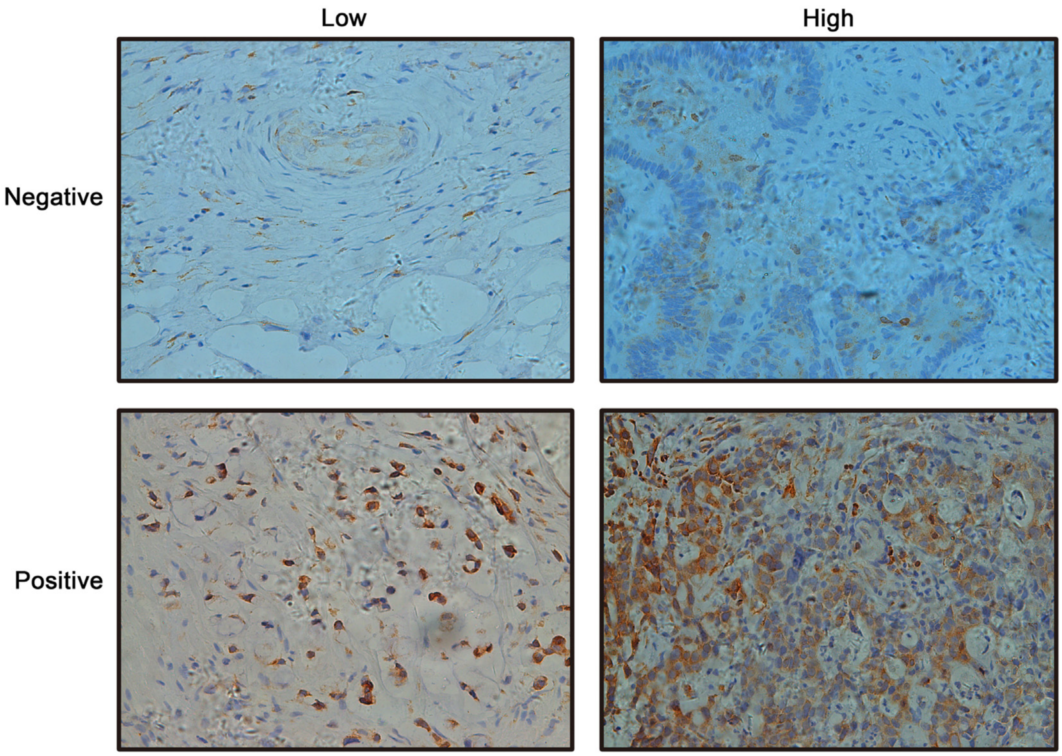

Supplementary Figure 1: Immunohistochemistry staining grade for RPN2. A total of 64 CRC specimens were classified into 2 groups based on RPN2 immunohistochemistry staining level: a positive RPN2 staining group (more than 10% of the cells were stained) and a negative RPN2 staining group (less than 10% of the cells were stained). The positive staining group was divided further into 2 groups according to the intensity of staining: high RPN2 staining (>50% of cells were intensely stained) and low RPN2 staining (<50% of cells were intensely stained).

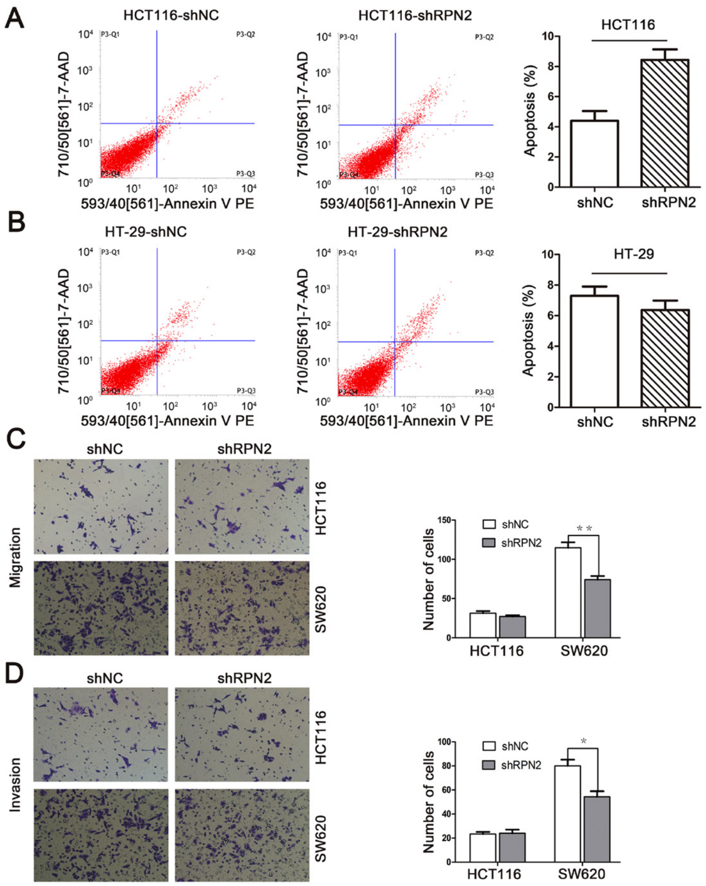

Supplementary Figure 2: Analysis of cell apoptosis, migration and invasion. (**A** and **B**) Effect of RPN2 on HCT116 and HT-29 cells apoptosis. Representative images of migration **(C)** and invasion **(D)** of HCT116 and SW620 cells transfected with shRPN2 and shRNA-NC are shown on the left panel (×200 magnification). The number of migrated and invaded cells was measured in the right panel, respectively, mean±SD, *, p<0.05. **, p<0.01.

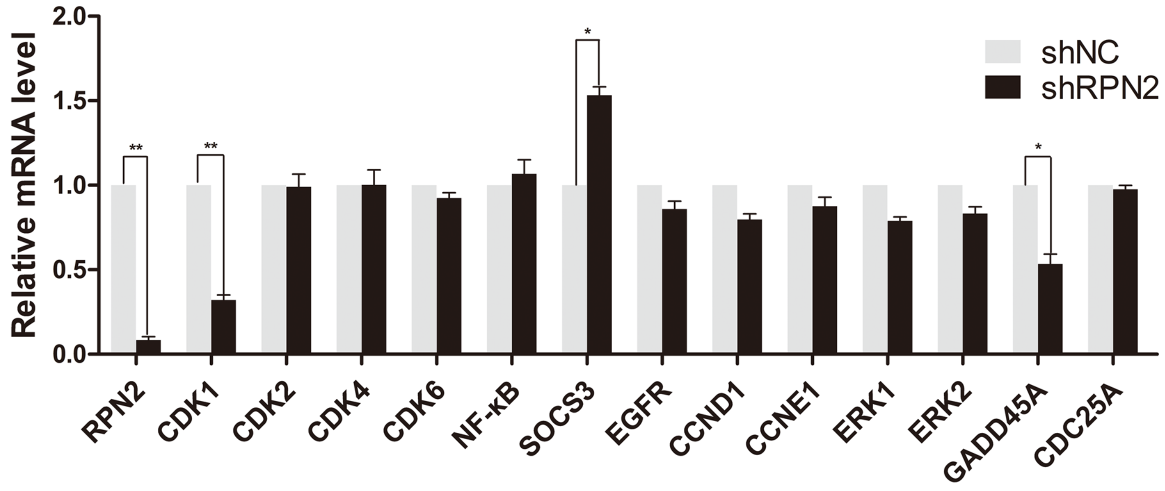

Supplementary Figure 3: The mRNA expression of some genes known to be critical for cell cycle. The mRNA levels of *RPN2, CDK1, CDK2, CDK4, CDK6, NF-κB, SOCS3, EGFR, CCND1, CCNE1, ERK1, ERK2, GADD45A*, and *CDC25A* were analyzed with qRT-PCR in HCT116-shRPN2 and HCT116-shNC cells. The results were shown as the mean±SD from three independent experiments. *, $p<0.05$. **, $p<0.01$.

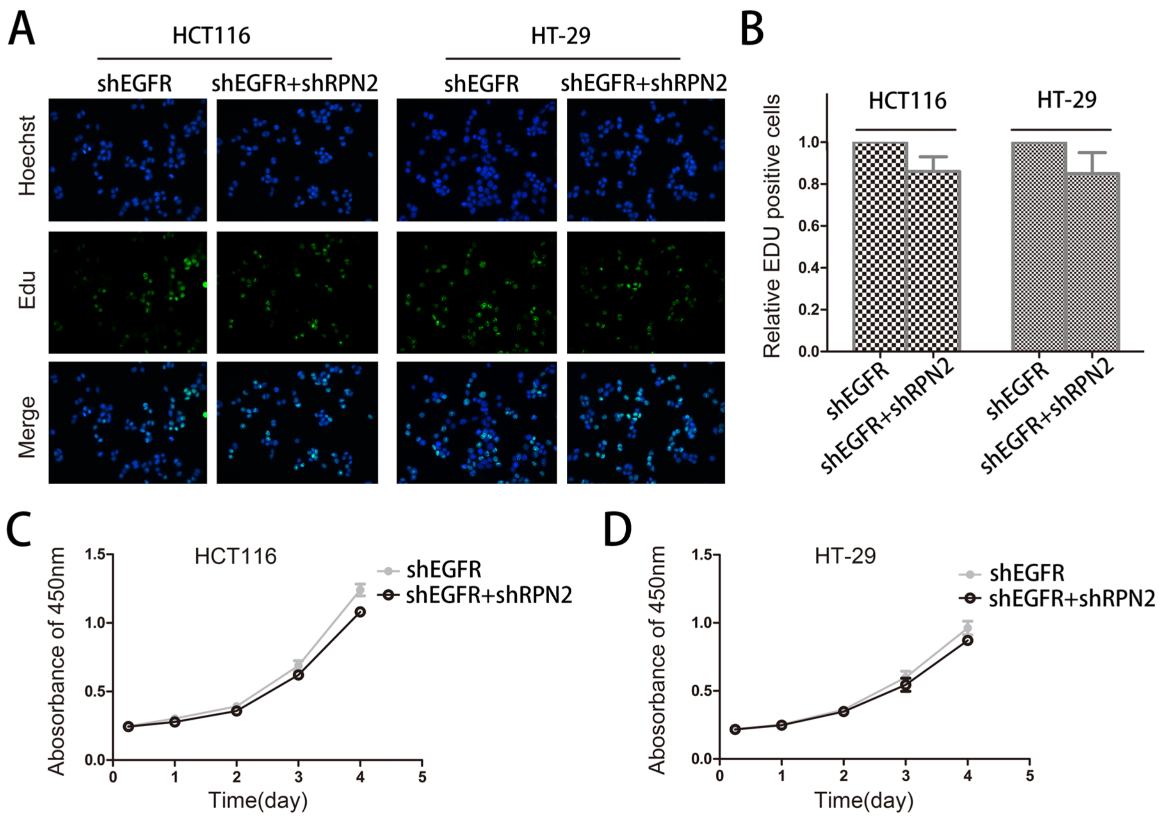

Supplementary Figure 4: Silencing RPN2 weakly inhibits CRC cell proliferation when EGFR is downregulated. (A and B) The effect of both EGFR and RPN2 silencing on the growth of colorectal HCT116 and HT-29 cancer cells compared with EGFR knockdown analyzed by EdU proliferation assay. **(C and D)** CRC cell proliferation was detected by CCK-8 and absorbance at 450 nm at different time-points is shown. Values at the indicated time-points represent mean±SD from three independent experiments.
